# Periodic limb movements in sleep are followed by increases in EEG activity, blood pressure, and heart rate during sleep

**DOI:** 10.1007/s11325-017-1476-7

**Published:** 2017-02-11

**Authors:** Mariusz Sieminski, Jan Pyrzowski, Markku Partinen

**Affiliations:** 1 Department of Adults’ Neurology, Medical University of Gdansk, Ul. Debinki 7, 80-952 Gdansk, Poland; 2 Vitalmed Helsinki Sleep Clinic, Valimotie 21, Helsinki, 00380 Finland

**Keywords:** Periodic limb movements, Blood pressure, Restless legs syndrome, Heart rate, Polysomnography

## Abstract

**Purpose:**

Periodic limb movements in sleep (PLMS) are related to arousal, sympathetic activation, and increases in blood pressure (BP), but whether they are part of the arousal process or causative of it is unclear. Our objective was to assess the temporal distribution of arousal-related measures around PLMS.

**Methods:**

Polysomnographic recordings of six patients with restless legs syndrome were analyzed. We analyzed 15 PLMS, plus three 5-s epochs before and after each movement, for every patient. Mean values per epoch of blood pressure (BP), heart rate (HR), and electroencephalographic (EEG) power were calculated. For each patient, six 5-s epochs of undisturbed sleep were analyzed as controls.

**Results:**

Alpha + beta EEG power, systolic BP, and HR were significantly increased following PLMS. The EEG power and HR increases were noticed in the first epoch after PLMS, whereas that of systolic BP was observed in the second and third epochs following a PLMS. No significant changes occurred in the epochs of undisturbed sleep.

**Conclusions:**

The results suggest that PLMS are followed by arousal-related nervous system events. Given the high frequency of PLMS throughout the night, they could be a potential risk factor for nocturnal arrhythmias and hypertension, in addition to causing sleep deprivation.

## Introduction

Periodic limb movements in sleep (PLMS) are repetitive, stereotypical involuntary movements of the lower extremities that appear during sleep. They typically consist of dorsiflexion of the toes and ankle, with partial flexion of knee and hip. A single movement may last from 0.5 to 10 s; however, the movements manifest in series of four or more single movements separated by intervals of 5 to 90 s [1]. They are highly prevalent in patients with restless legs syndrome (RLS) [2], but are also found in patients with narcolepsy [3], REM sleep behavior disorder [4], and insomnia [5]. Some PLMS are followed by arousals, defined as transient increases in higher frequency (fast) electroencephalographic (EEG) activity occurring with increases of sympathetic activity. These are called periodic limb movements with arousals (PLMA).

PLMS are followed by a significant increase in heart rate (HR) and blood pressure (BP), which is even greater following PLMA. This phenomenon has been described in patients with restless legs syndrome (RLS) [6, 7] and in healthy subjects [8].

The nature of the relationship between PLMS and sympathetic activation, whether causal (and the direction of causality) or associative, is not clear. Pharmacological suppression of PLMS does not eliminate the arousals, and suppression of the arousals does not diminish the appearance of PLMS [9, 10]. Experimental arousals evoked during sleep do not increase the number of PLMS [11]. These results suggest that PLMS may appear with arousal (as PLMS with arousal, PLMSA) but also both phenomena may appear independently.

The most intuitive hypothesis is that PLMS cause the arousals. An alternative explanation, though, is that PLMS are just another symptom of generalized arousal during sleep. There is a third possibility, that PLMS appear coincidentally during unstable sleep characterized by the presence of cortical and autonomic arousals.

The aim of this study was to analyze the temporal distribution of changes in EEG activity, BP, and HR that precede and follow a PLMS, and to compare this to the course of such changes during sleep uninterrupted by leg movements. There are four imaginable situations: (1) PLMS cause arousal—then changes in those parameters would appear after the movement; (2) arousal evokes PLMS—then the changes would be detectable before the movement; (3) PLMS are coincidental with spontaneous arousals—then signs of activation should be present during sleep without any leg movements; and (4) arousals and PLMS share a common underlying factor, which evokes signs of arousal detectable by surface recordings and PLMS with different (and potentially variable) time lags.

Understanding this relationship has therapeutic implications. For example, if EEG arousal and BP increase are evoked by PLMS, reducing the number of PLMS (e.g., with dopaminergic therapy) could potentially reduce nocturnal BP, and thus nocturnal hypertension, in patients. If the causal relationship is in the opposite direction, nocturnal hypertension would need to be treated with sympatholytics. If arousals and PLMS are evoked by the same unknown factor then new models of experiments should be developed to determine the nature of such a factor.

## Methods

The study protocol was approved by the Independent Bioethical Committee for Scientific Research at the Medical University of Gdansk.

### Subjects

Six subjects with idiopathic RLS were recruited for polysomnographic (PSG) analysis. Subjects were fully anonymized after their initial selection. The patients were diagnosed with RLS according to International Restless Legs Syndrome Study Group criteria [12]. All the patients participating in the study underwent a neurological examination allowing exclusion of RLS mimics, such as polyneuropathy or nocturnal cramps. Patients also underwent a structured interview performed by a sleep specialist during which data on sleep-related signs and symptoms were collected. This interview allowed to exclude the presence of sleep disorders other than RLS. The following inclusion criteria were used: Patient’s signed agreement to undergo PSG and to analyze their data for scientific purposes; patients were diagnosed with RLS according to International Restless Legs Syndrome Study Group criteria [12] and underwent a full PSG with continuous beat-to-beat BP measurements; the periodic limb movements in sleep index (PLMSI) was above 15. The exclusion criteria were as follows: presence of any co-morbid sleep disorder; presence of sleep-related breathing disorders, defined as an apnea-hypopnea index (AHI) greater than 5; presence of any psychiatric disorders, especially mood disorders; usage of psychotropic drugs; diagnosed hypertension; presence of any cardiovascular events in history, e.g., acute coronary syndrome or stroke; intake of any drugs that may interfere with the autonomic nervous system or cardiovascular system, especially antihypertensive or antiarrhythmic drugs; current (in the 2 weeks preceding PSG) pharmacological treatment for RLS, and presence of augmentation actually or in the history.

### Recordings

Patients underwent a single PSG study. All recordings were performed with the SOMNOscreen plus PSG system (Somnomedics, Randersacker, Germany). Sleep recordings included four EEG leads, two bilateral electro-oculogram leads (EOG), bilateral chin electromyographic leads (EMG), and two surface EMG leads placed on the left and right anterior tibialis muscles (for recording periodic limb movements). Respiration was recorded with a nasal cannula, thoracic and abdominal strains, and finger oxymetry. Electrocardiograms were recorded with a single precordial lead. The PSG included beat-to-beat blood pressure measurement determined using pulse transit time (PTT) [13]. The measurement of blood pressure was continuous, non-invasive, and did not disturb the sleep of the patients. The PTT-based measurement of BP was calibrated and then validated against sphygmomanometric (cuff) measurement of BP on the brachial artery at the beginning of the recording.

Electromyographic recordings were performed with surface electrodes placed bilaterally on the anterior tibialis muscles. For recording from each leg, a separate channel was used.

Periodic leg movements were recognized when an increase of at least 8 μV above the resting line in EMG appeared and lasted for 0.5–10 s before a drop in EMG to <2 μV above the resting line. The episodes were considered as PLMS only when they appeared in series of at least four such episodes separated by intervals lasting from 5 to 90 s.

Leg movements overlapping with any breath event (e.g., apnea) were not considered as PLMS. Leg movements appearing sooner than 0.5 s before the beginning or no more than 0.5 s after the end of a breath event were not considered as PLMS.

The PSG recordings were scored according to American Academy of Sleep Medicine guidelines [14]. The following sleep parameters were calculated: total sleep time (TST); sleep efficiency (SE); latency of stages 1, 2, slow wave sleep (SWS), and REM sleep; duration of stages 1, 2, SWS, and REM; sleep stage change index (number of transitions between the sleep stages per hour of sleep); the wake index (WI; number of awakenings per hour of sleep), duration of wake after sleep onset (WASO), and PLMS index (PLMSI).

### Selection of PLMS and normal sleep periods

There were 15 single periodic leg movements (PLM) selected for each patient. Only movements appearing during stage 2 sleep were selected to avoid the potential confound of sleep stage differences. Each PLM was taken from a series of PLMS defined according to WASM/IRLSSG criteria [1]. Each selected PLM was separated from the previous and next individual movements with a gap of at least 20 s, to avoid any interference from effects related to the adjacent movements. We have selected only leg movements that were not interfering with any other physiologic phenomenon (e.g., any disorders of breathing, shifts of sleep stage) or with technical artifacts.

For control epochs, ten 30-s long periods of undisturbed stage 2 sleep were selected for analysis for each patient. Control periods were free of any leg movements, respiratory events, or artifacts.

### Analysis

**Fig. 1:**
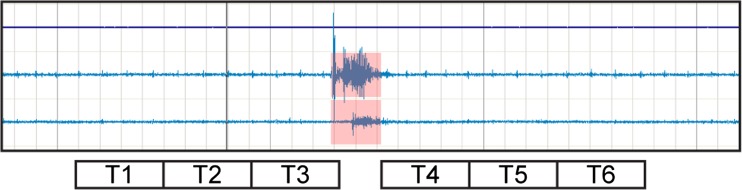
Epochs selected for analysis. For each epoch, mean values of systolic BP (SBP), diastolic BP (DBP), and heart rate (HR) were analyzed. For analysis of changes of electroencephalographic (EEG) activity, EEG power was calculated using fast Fourier transformation (FFT) for alpha + beta and delta bands, then calculating the means for each band per 5-s epoch. The calculations were performed with DOMINO (Somnomedics, Randersacker, Germany) analysis software

The 15-s long periods before and after each selected movement were divided into 5-s epochs named T1, T2, and T3 (epochs before the movement) and T4, T5, and T6 (epochs after the movement) (Fig. 1).

Analyzing 5-s epochs allowed us to verify if there is any prolonged effect of the PLMS, lasting longer that a series of heart evolutions.

The 30-s long periods of undisturbed sleep were also divided into 5-s long epochs named consecutively from T1 to T6.

For each epoch, the following physiological parameters were obtained: alpha + beta (1) and delta (2) EEG power (calculated with the use of standard fast Fourier transform (FFT) tools), average heart rate (3), average systolic (4), and diastolic (5) blood pressure values (measured continuously beat-to beat).

### Statistical analysis

The patterns of peri-PLMS fluctuations of the studied physiological parameters were assessed using 2-way ANOVA. Every six-element time series (associated with a single PLMS event) was normalized by subtraction of its temporal mean. The first independent variable was the time index within the time series (a fixed effect). As multiple events were studied for each patient, the second independent variable was chosen to be the patient index (a random effect). The presence of a significant peri-PLMS variation pattern was assumed to correspond to (1) the significance of the time index-related effect, together with (2) the insignificance of the patient index-related effect (indicating that the pattern is present for all patients). The statistical model also included the interaction between the two independent variables, which may be considered to represent patient-specific variability in fluctuation magnitude. Analysis was performed analogously for every studied parameter and then repeated for the surrogate event group. Values of *p* less than or equal to 0.05 were considered statistically significant. Post hoc multiple comparisons between individual epochs were performed according to Tukey’s procedure. Statistical analysis was performed with Matlab software (Mathworks, 2014).

#### Results

PSG recordings of six subjects were included for analysis. Their demographic features and sleep characteristics are presented in Table 1. Mean total sleep time (TST) was 306.4 min, with noticeably low mean sleep efficiency (59.3%). The study population was also characterized by a long wake time after sleep onset (WASO), as much as 3 h. The mean number of periodic limb movements in sleep per hour of sleep (PLMS index) was high (57.9), which would be expected in a population of patients with RLS.

**Table 1:** Demographics and sleep characteristics of patients

Gender (M/F)	3/3
Age at PSG	57.0 ± 22.1
BMI (kg/m^2^; mean ± SD)	27.8 ± 4.0
Total sleep time (minutes; mean ± SD)	306.4 ± 116.2
Sleep efficiency (%; mean ± SD)	59.3 ± 20.3
Seep latency (minutes; mean ± SD)	32.6 ± 29.1
WASO (minutes; mean ± SD)	169.4 ± 107.4
Awakenings index (mean ± SD)	6.9 ± 3.8
Arousal index (mean ± SD)	11.5 ± 15.0
Sleep stage change index (minutes; mean ± SD)	14.3 ± 3.3
Sleep stage % (mean ± SD)	
REM (% of total sleep time)	15.1 ± 7.6
1 (% of total sleep time)	18.0 ± 7.4
2 (% of total sleep time)	47.8 ± 3.9
SWS (% of total sleep time)	18.5 ± 6.9
PLMS index	57.9 ± 27.3
PLMW index	25.3 ± 11.5
PLMA index	5.6 ± 4.8
SBP during night (mmHg; mean ± SD)	126.8 ± 19.8
DBP during night (mmHg; mean ± SD)	77.8 ± 15.9
HR during night (mean ± SD)	58.3 ± 9.5

*PSG* polysomnography, *SD* standard deviation, *BMI* body mass index, *WASO* wake after sleep onset, *SWS* slow wave sleep, *PLMS* periodic limb movements in sleep, *PLMW* periodic limb movements in wake, *PLMA* periodic limb movements with arousal, *SBP* systolic blood pressure, *DBP* diastolic blood pressure, *HR* heart rate

The mean values of the analyzed parameters per epoch are presented in Table 2. A significant increase of alpha + beta wave power (*p* = 0.05), systolic blood pressure (*p* < 0.001), and heart rate was found following PLMs (*p* < 0.001). The significant increases in alpha + beta EEG power and heart rate were noticed in first 5 s following the movement (the T4 epoch). The increase in systolic blood pressure was first observed in epoch T5 (5 to 10 s following a movement), and the increase persisted through T6. There were no significant changes in the measured values in epochs preceding the PLM (T1 to T3); nor were there any significant changes in the values of diastolic blood pressure and delta EEG power.

**Table 2:** Mean values of analyzed parameters per epoch.Values of parameters that are significanlty different from the preceding ones are marked with italics.

	T1	T2	T3	T4	T5	T6	*p*
PLMS							
SBP (mmHg; mean ± SD)	127.4 ± 17.2	126.7 ± 17.5	125.8 ± 17.7	125.5 ± 17.0	*128.8 ± 17.8*	*128.8 ± 17.8*	<0.001
DBP (mmHg; mean ± SD)	79.8 ± 13.4	79.8 ± 13.4	79.4 ± 13.3	79.3 ± 13.3	79.3 ± 13.3	79.5 ± 13.3	NS
HR (bpm mean ± SD)	58.1 ± 9.3	57.7 ± 9.3	58.9 ± 8.8	*63.1 ± 7.7*	58.3 ± 10.4	57.3 ± 9.6	<0.001
A + B FFT (mean ± SD)	49.1 ± 11.1	48.9 ± 10.8	49.2 ± 11.4	*50.8 ± 12.4*	50.3 ± 11.7	49.0 ± 10.1	0.05
D FFT (mean ± SD)	31.1 ± 7.7	31.1 ± 7.6	31.3 ± 8.1	30.5 ± 8.5	30.2 ± 8.2	31.0 ± 7.7	NS
No PLMS							
SBP (mmHg; mean ± SD)	124.4 ± 16.9	124.4 ± 17.0	124.3 ± 16.9	124.1 ± 16.9	124.3 ± 16.3	124.9 ± 16.6	NS
DBP (mmHg; mean ± SD)	79.2 ± 14.2	79.2 ± 14.1	79.4 ± 14.2	79.3 ± 14.3	79.4 ± 14.2	79.4 ± 14.2	NS
HR (mean ± SD)	57.5 ± 9.0	57.7 ± 9.9	57.1 ± 9.6	57.1 ± 9.3	58.0 ± 9.7	57.5 ± 10.7	NS
A + B FFT (mean ± SD)	51.8 ± 9.6	52.2 ± 9.6	51.8 ± 9.3	51.2 ± 9.7	50.9 ± 9.3	51.6 ± 9.8	NS
D FFT (mean ± SD)	29.7 ± 6.1	29.4 ± 6.5	29.7 ± 6.4	30.1 ± 6.9	30.0 ± 6.8	30.0 ± 6.9	NS

*A + B* alpha + beta wave power, *BPM* beats per minute, *DBP* diastolic blood pressure, *D* delta wave power, *FFT* fast Fourier transform, *HR* heart rate, *PLMS* periodic leg movements in sleep, *SBP* systolic blood pressure, *SD* standard deviation

No significant changes in any of the analyzed parameters were observed for the consecutive epochs of undisturbed sleep.

**Fig. 2:**
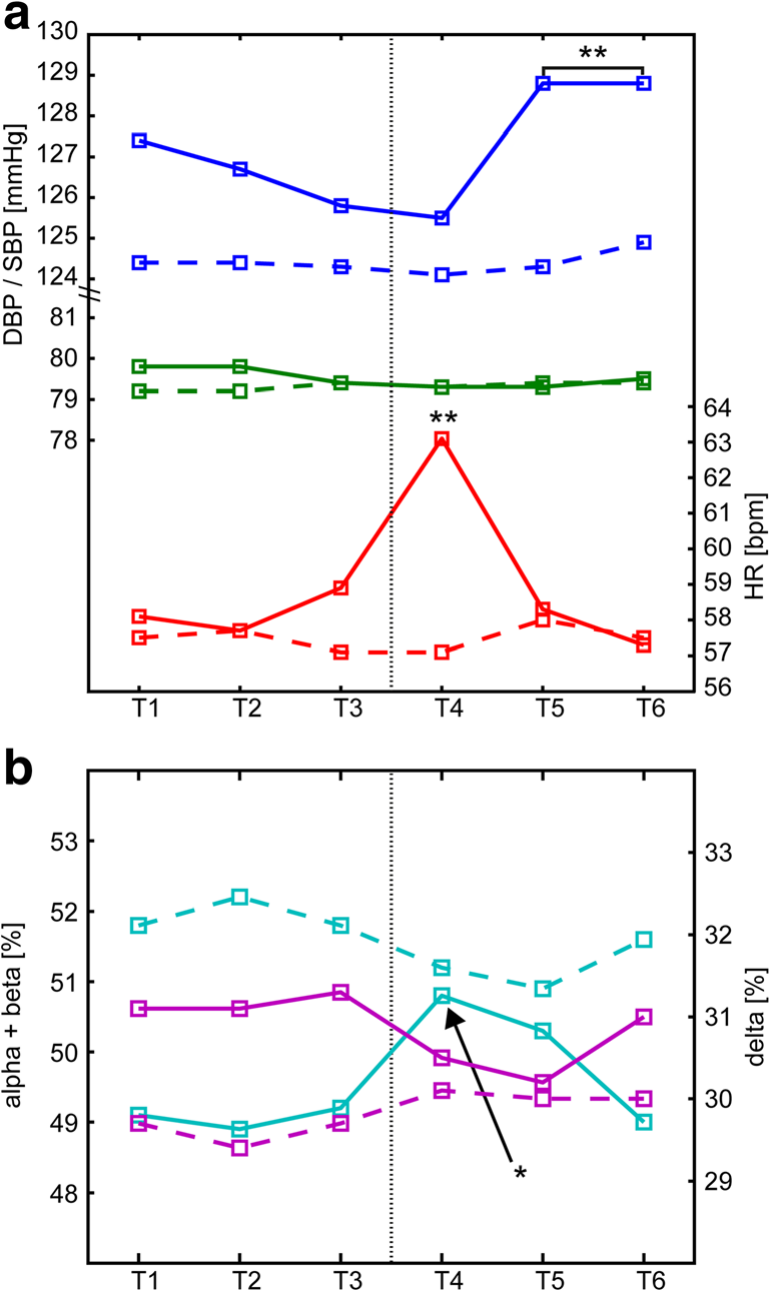
Graphical illustration of peri-periodic limb movements in sleep (PLMS) fluctuations of the studied parameters. **a** Cardiovascular parameters: significant fluctuations are present for systolic blood pressure (*SBP*) and heart rate (*HR*) in the PLMS-related epochs (*solid blue* and *red lines*), while no significant fluctuations are present for diastolic blood pressure (*DBP*, *green solid line*) or for periods of undisturbed sleep (*dashed lines*). *Stars* indicate epochs that differed significantly from all other epochs following Tukey’s multiple comparisons tests. **b** Electroencephalography (EEG) parameters: A significant increase in alpha + beta power is noticeable directly after the PLMS event (*blue solid line*, *star*), without significant fluctuations in delta power (*purple solid line*), and for periods of undisturbed sleep

The results demonstrate a temporal sequence to the phenomena, such that the leg movement (PLM) immediately preceded cortical activation, which was accompanied by an increase in heart rate. The cortical activation was immediately followed by the increase in blood pressure. Changes of parameters are shown in Fig. 2.

## Discussion

Our results show a temporal relation between PLMS and cortical activation, heart rate and blood pressure. Appearance of PLMS is followed by a significant increase in alpha + beta wave power and heart rate, and then shortly thereafter by an increase in systolic blood pressure. We did not detect any changes in EEG, BP, and HR preceding the PLMS. Analysis of periods of sleep without any events also showed no changes in those parameters. That suggests that the changes accompanying PLMS differ qualitatively and quantitatively from those typically appearing during normal sleep.

PLMS may be a potent factor alternating the status of a sleeping subject and thus having an impact on his daytime functioning. This impact may be reflected by functions of vascular system or sensory systems. A good example of such interplay is migraine: a neurologic disease with disturbed vascular function and processing of sensory/painful stimuli. Prevalence of RLS/PLMS is higher in subjects with migraine (both adults and children) what suggests a link between the presence of PLMS and disordered control over vascular and sensory processes [15, 16].

Autonomic and cortical activity related to PLMS has been the object of analysis in the past. Sforza et al. found that PLMS are followed by tachycardia (shortening of the R-R interval on electrocardiographic recordings), which starts immediately after the onset of the leg movement and lasts for three beats after the PLM [17]. The authors also found that PLMS are followed by a significant increase in EEG activity. These findings are concordant with our observations of cortical activity and HR increasing significantly in the first 5 s after the PLM. Those findings also led Sforza et al. to propose a cascade of responses to PLMS, starting with autonomic activation, followed by EEG changes and a subsequent arousal (awakening) [17]. Our results confirm this hypothesis. Winkelman et al. described a similar phenomenon—an increase in HR following a PLM [18]. Notably, the heart rates of the patients in the above study were highest for the first eight beats following the PLM, which is timed similarly to the HR changes found in our study. However, researchers have also found in that study that HR drops just before the PLMS, though our study cannot confirm this. The fact that PLMS are followed by tachycardia (with a peak value at fifth to sixth cardiac evolution after the PLMS) was also shown by Gosselin et al. [19]. The authors of that study also reported that the magnitude of that reaction depended on the age of the patients, but that no significant changes in heart rhythm preceded the PLMS [19]. Like Sforza and colleagues, Winkelman et al. postulated that PLMS produce arousals associated with autonomic changes [18]. Both the above studies and our data support the proposed hypothesis that PLMS induce arousal, which manifests as both brain and autonomic activation.

Pennestri et al. published evidence that PLMS are related to a significant increase in blood pressure in patients with RLS. They analyzed HR and BP directly after a PLM, finding a significant increase in HR (with a peak value at the fifth beat after the PLMS) and in BP (peaking at the ninth heart beat) [7]. Their results and those from other teams regarding healthy patients [6–8] indicate an increase in sympathetic activity following a PLM. Indeed, all of the cited studies, as well as the data presented herein, demonstrate that the changes in HR and BP following PLMS are very consistent.

Despite this consistency in the data, our results contained one notable exception: We did not observe a significant increase in diastolic blood pressure. However, the increase in DBP found by the teams cited above [6–8] was much smaller than the increase in SBP. Furthermore, it became visible when analyzing single cardiac evolutions. Given these differences, our use of averaging values over 5-s epochs could inadvertently mask such changes.

Our results show that cortical and autonomic activation starts after the PLMS. However, there are studies demonstrating that some forms of activation begin before the PLMS, and that the leg movements are part of an ongoing process of activation. For example, Ferillo et al. published an article showing an increase in EEG activity just before the PLMS—about 3 s before for delta band activity and 1 s before for the other EEG bands. Notably, the authors also found an increase in HR 4 s before a PLMS [20]. Ferri et al. observed an increase in HR appearing 1 s before the start of PLMS and an increase in EEG power appearing about 7–8 s before the onset of PLMS [21]. Sasai et al., in an observational study of patients with periodic limb movement disorder (PLMD: clinically significant insomnia and/or hypersomnia without any other cause than the presence of PLMS), found that there is a difference in heart rate variation (HRV) between sleep periods with and without PLMS, suggesting that sympathetic activity is dominant in periods with PLMS. Moreover, changes in HRV heralding the beginning of a PLMS-containing sleep period start long before the onset of the first PLM [22].

We found no changes in any of the measured parameters preceding the PLMS. Those changes described by the authors cited above [20, 21] were relatively small and short-lived; therefore, they may have been masked by our averaging method, just as we believe the diastolic BP changes noticed by other studies [6–8] were. The largest increases of HR, EEG power, and BP were uniformly noticed after the PLMS by all authors. We also found no significant changes of measured values of HR, BP, or EEG power in undisturbed (e.g., no leg or respiratory events) sleep. This provides additional support for hypothesis that the PLMS precede cortical and autonomic arousal. Nevertheless, it cannot be ruled out that such changes, including PLMS, are simply an element of continuous fluctuations in physiological parameters during sleep. The significance of such fluctuations is difficult to establish with the available data. The hypothetical factor responsible for rhythm and amplitude of such fluctuations remains a theoretical concept. Attempts to verify this concept by analyzing coincidence of various changes of physiological parameters would bring us new interesting data on central control over autonomic functions of the human body.

Our study has certain limitations. One is inherent in our method of analyzing the PSG recordings. We calculated mean values for 5-s epochs, in order to detect sustained changes with a prolonged effect. However, this limited our ability to identify short and/or small changes in the parameters. The study was also limited by the number of analyzed events. The criteria used for their selection certainly reduced the number of acceptable events, though they did allow us to limit some confounding factors and create a pool of homogeneous events for analysis. Nevertheless, future projects should enlarge the number of events to better clarify these relationships. This study also utilized a relatively small number (six) of patients, all of whom had RLS. Both of these conditions reduced our ability to generalize to a broader population, such as those with other sleep disorders. We chose to restrict the population to those with RLS because the high number of PLMS common in RLS could cause this population to be at a higher risk of cardiovascular disturbances. Expanding this analysis to include healthy subjects would add additional data about the role of PLMS in nocturnal changes in EEG and cardiovascular activity. Despite these limitations, our protocol provided a non-invasive, non-disturbing method of measuring BP in sleep. It enabled us to collect very precise, beat-to-beat values of BP without significant interference or experimentally induced arousal (i.e., false positives). Our criteria eliminated confounders such as comorbidities and differences in sleep stage, and our analysis of undisturbed sleep in the same group provided an internal control. Together, these conditions support our hypothesis that the observed variations of the parameters can be temporarily related to PLMS.

In conclusion, our study allowed the detection of patterns of changes in sleep parameters related to PLMS. We have described a temporal relation, not a causal one; nevertheless, our findings make suggestion that PLMS evoke activation in the brain, accompanied by a sympathetic activation, worthy consideration. As this sequence is repeated many times during the night, it might be considered a potential risk factor for nocturnal arrhythmias and nocturnal hypertension, increasing the risk of cardiovascular problems in the patients.
